# Diversity of Phytosterols in Leaves of Wild Brassicaceae Species as Compared to *Brassica napus* Cultivars: Potential Traits for Insect Resistance and Abiotic Stress Tolerance

**DOI:** 10.3390/plants12091866

**Published:** 2023-05-01

**Authors:** Muhammad Burhan Bootter, Jing Li, Wenxu Zhou, David Edwards, Jacqueline Batley

**Affiliations:** School of Biological Sciences, University of Western Australia, Perth, WA 6009, Australia

**Keywords:** phytosterols, phytosterol diversity, insect resistance, abiotic stress tolerance, crop improvement, breeding strategy, *Brassica napus*

## Abstract

Phytosterols are natural compounds found in all higher plants that have a wide variety of roles in plant growth regulation and stress tolerance. The phytosterol composition can also influence the development and reproductive rate of strict herbivorous insects and other important agronomic traits such as temperature and drought tolerance in plants. In this study, we analysed the phytosterol composition in 18 *Brassica napus* (Rapeseed/canola) cultivars and 20 accessions belonging to 10 related wild Brassicaceae species to explore diverse and novel phytosterol profiles. Plants were grown in a controlled phytotron environment and their phytosterols were analysed using a saponification extraction method followed by GC-MS from the leaf samples. The *B. napus* cultivars showed slight diversity in eight phytosterols (>0.02%) due to the genotypic effect, whereas the wild accessions showed significant variability in their phytosterol profiles. Of interest, a number of wild accessions were found with high levels of campesterol (HIN20, HIN23, HUN27, HIN30, SARS2, and UPM6563), stigmasterol (UPM6813, UPM6563, ALBA17, and ALBA2), and isofucosterol (SARS12, SAR6, and DMU2). These changes in individual phytosterols, or ratios of phytosterols, can have a significant implication in plant tolerance to abiotic stress and plant insect resistance properties, which can be used in breeding for crop improvement.

## 1. Introduction

Phytosterols are isoprenoid compounds produced by the mevalonate pathway in all plants [1]. There are about 40 different phytosterols that are commonly produced in higher plants, as characterised in *Arabidopsis thaliana* [2], whereas more than 200 phytosterols have been found in plant species [3]. Common predominant phytosterols are sitosterol, campesterol, and stigmasterol, while others can be found in lesser amounts or are intermediate structures [4]. The accumulation of these phytosterols can affect plasma membrane properties, growth regulation, and stress responses [5,6,7]. Recently, there has been a spike in phytosterol studies from the roots and leaves due to their potential application for crop improvement; these include temperature tolerance [8], salt tolerance [9], drought tolerance [10], pathogenic bacterial attack [11], and insect resistance [12]. Analysing phytosterol variation due to genotypic effects in plants can assist in the introgression of phytosterol related traits into commercial cultivars through breeding.

*Brassica napus* (rapeseed/canola) is the third largest oilseed crop globally, accounting for about 20% of the world’s oilseed production [13]. It is mainly used for producing edible oil and animal fodder. *B. napus* also serves in the production of motor engine lubricant, lamp fuel, biodiesel, and as ingredients in soap and margarine [14], whilst related wild Brassicaceae species are used for edible roots and condiments [15]. In *B. napus*, the phytosterol profile studies performed to date have been mainly focused on oil seed samples as phytosterols have a wide variety of health advantages, mainly known for lowering cholesterol absorption [16] and anti-inflammatory activity [17]. So far, the phytosterol composition variability of leaves or other functional parts of the plant in *B. napus* genotypes has not been analysed.

Particular phytosterol composition in leaves can bring resistance against targeted herbivorous insects by hindering insect growth and development [18]. Insect sterol metabolism is not well-adapted to all types of phytosterols present in plants [19]; they require strict phytosterol structures as substrates for their cholesterol synthesis, and such requirements differ based on the insect species [20]. For example, species belonging to the order Orthoptera have shown weak or no growth when fed on stigmasterol whereas other insect species such as *Blattella germanica,* belonging to the order Dictyoptera, thrive on stigmasterol [20]. Phytosterols are a micronutrient needed by insects in a small amount, however, in a mixed phytosterol diet, if the composition of unsuitable phytosterols is increased, less suitable phytosterol is utilized by insects. For example, two insect species, *Heliothis virescens* and *Helicoverpa zea,* were fed on artificial mixed diets with different ratios of suitable and unsuitable sterols. In a diet with a high ratio of unsuitable sterol, even with enough of the suitable sterol present, the insects had a lower larval development rate, pupal mass, eclosion success, and egg production [21]. Similarly, a recent study using transgenic *A. thaliana* lines, where the unsuitable phytosterol ratio was increased, the aphids (*Myzus persicae*) that fed on this had a lower growth mass and pupation rate [12]. This suggests that crop cultivars containing a high ratio of unsuitable to suitable phytosterols in their leaves or phloem have a potential of having a higher insect resistance.

Recent functional studies have shown that plants can become tolerant to abiotic stresses by having set phytosterol ratios in their functional parts such as roots and leaves. Increasing a particular sterol ratio affects the state of the plasma membrane, thereby improving the plant’s ability to deal with environmental stresses such as pathogen attack, drought, salinity, and temperature [7]. It is speculated that raising the stigmasterol to sitosterol ratio affects a cell membrane’s flexibility, integrity, and fluidity [22]. In transgenic *A. thaliana*, an increased interconversion of stigmasterol from sitosterol improves the cell membrane structure, causing less leakage in extreme temperature conditions and making plants more temperature tolerant [8]. Moreover, it has been reported that elevated stigmasterol levels in plants results in enhanced resistance to bacterial pathogen infection [11] and enhanced drought tolerance [23].

In two previous phytosterol screening studies, *B. napus* seed samples were used to analyse the phytosterol composition between genotypes [24,25]. These studies included 19 and 27 *B. napus* commercial cultivars grown in one and six different field conditions, respectively, and reported high variability between genotypes in major phytosterols [24,25]. However, environmental conditions and planting locations have an effect on phytosterol composition [26]. In the Brassicaceae family, analysis of the phytosterol profile from leaf has been reported from *A. thaliana* [27], whereas the variation between genotypes in leaf samples grown in a controlled environment is limited to Chinese cabbage (*Brassica rapa* L. subsp. *Pekinensis*) [28].

Phytosterol composition in plants is a complex trait, and the exact mechanisms and genetic factors affecting the individual phytosterols and ratios are still unclear. So far, two genome wide association studies have been conducted to find the genotypic and environmental effects [29,30]. Both studies analysed *B. napus* commercial cultivar seed samples grown in field conditions and found significant phytosterol variation due to genotypic and environmental effects. In an earlier study [29], between four and eight quantitative trait loci (QTL) were detected for individual phytosterols (sitosterol, campesterol, brassicasterol, and avenasterol), together explaining between 48 and 68% of the total genetic variance of the respective trait. However, no candidate genes were identified. In the later study [30], between one and six QTL were detected for each phytosterol, revealing 88, 66, 74, and 34% of the genetic variance for sitosterol, campesterol, brassicasterol, and avenasterol, respectively. For the sitosterol to campesterol ratio, a major QTL overlapped with the sterol methyltransferase 2 (SMT2) gene and another QTL, associated with brassicasterol, overlapped with the C22-sterol desaturase 1 (CYP710A1) gene. For the rest of the QTLs, no other candidate genes were suggested. However, these studies showed a high genetic effect contributing to the phytosterol variation observed with plants grown in field conditions for a whole crop cycle [29,30]. This indicates that genetic factors can be used for breeding to achieve the ideal sterol compositions in crops. Moreover, more phytosterol screening studies need to be performed to identify novel genotypes for phytosterol traits.

Finding wide variation in phytosterol profiles among genotypes can be the key to acquiring the ideal phytosterol profiles in plants through molecular marker assisted breeding [31,32,33]. However, *B. napus* commercial cultivars have been suggested to have a reduced overall genetic diversity due to thousands of years of domestication, and being under constant artificial selection pressure for increasing nutritional value, this process has led to plants with weak defences [31,32,34]. Wild accessions can be a source of novel traits such as insect resistance, rare phytosterols, and novel phytosterol profiles. Studies on Brassicaceae wild species have revealed that there are wide varieties of species showing resistance to insect pests [32,35]. For example, *Brassica fruticulosa* has different levels of resistance to cabbage root fly (*Delia radicum)* [36,37] as well as to the cabbage stem weevil (*Ceutorhynchus pallidactylus*) [38], mustard aphid (*Lipaphis erysimi*) [39], and cabbage aphid (*Brevicoryne brassicae*) [40,41]. *Sinapis arvensis* was resistant to the large white butterfly (*Pieris brassicae* L.) [42,43] whereas *Diplotaxis muralis* and *Hirschfeldia incana* showed strong resistance against mustard aphid *(L. erysimi*) [44]. Furthermore, *Sinapis alba* has been identified to date as the most versatile species conferring resistance to a number of insect pests such as the cabbage seed weevil (*C. chusassimilis*), the pollen beetle (*Brassicogethes aeneus)*, and Crucifer flea beetle (*Phyllotreta cruciferae*) [32,35]. Several other wild accessions of *Brassica montana* and *Brassica macrocarpa* showed medium to high levels of tolerance to cabbage root fly (*D. radicum*) [37] and cabbage whitefly (*Aleyrodes proletella*) [45]. These examples provide evidence that wild species possess insect resistant properties, although the mode of action may differ, so a novel sterol profile in those wild accessions could represent one of the underlining mechanisms as detailed insect feeding studies have shown insects rendered on wild Brassicaceae accessions have a delayed developmental period and smaller adult mass compared to cultivated accessions [43,46,47]. Unsuitable phytosterol profiles can be a factor affecting the growth and development of insects [21], however, Brassicaceae wild accessions have not been analysed for phytosterol composition.

Currently, *B. napus* phytosterol studies only exist in oilseed samples grown in field conditions, not in leaf tissues, unlike in a Brassicaceae family member, Arabidopsis, where phytosterol composition similarities between the seeds and leaf tissues have been established [27]. However, the main emphasis of this study was to examine leaf phytosterols grown in controlled environmental conditions to investigate the genotypes’ phytosterol related insect and temperature tolerance capabilities. In addition, we aimed to explore genotypes with rare phytosterol profiles that could prove useful for devising strategies to achieve key phytosterol ratios in the leaf for agronomic traits. In this study, we profiled leaf samples taken from *B. napus* commercial cultivars and related wild Brassicaceae accessions and discovered novel individual phytosterol compositions and phytosterol ratios in wild accessions. We also discuss the potential application of sterol profile modification for crop improvement.

## 2. Results

Eight predominant phytosterols (>0.02% of the total phytosterol) were found in the leaf samples of the *B. napus* commercial cultivars (Cm) and related wild Brassicaceae accessions (Wl). Sitosterol was found to be the most abundant sterol followed by campesterol (Table 1). There were highly significant (*p* < 0.01) variations observed between *B. napus* commercial and wild Brassicaceae genotypes for sitosterol, isofucosterol, and stigmast-7-enol, whereas it was significant (*p* < 0.05) for 24-methylcholesterol, campesterol, sitostanol, and stigmasterol, and non-significant (*p* > 0.05) for cholesterol. There was a wider range and higher variation (SD) observed for all eight phytosterols among the Brassicaceae wild accessions compared to the *B. napus* commercial cultivars (Table 1). No *B. napus* commercial cultivar was found to have a unique phytosterol profile (Table 2) whereas wild accessions presented several novel phytosterol profiles (Table 3).

### 2.1. Novel Phytosterol Profiles in Wild Brassicaceae Accessions

High individual compositions of campesterol, isofucosterol, and stigmasterol were found in the wild accessions. Four wild accessions, UPM 6813 (*B. montana*), UPM6563 (*Brassica incana*), ALBA17 (*S. alba*), and ALBA2 (*S. alba*), had significantly higher stigmasterol levels at 2.65, 3.20, 4.55, and 9.75%, respectively, in comparison to *B. napus* commercial cultivars with 0.95 ± 0.25%. In these four wild accessions, a higher stigmasterol content seemed to be at the expense of sitosterol (Table 3). Three wild accessions, SARS12 (*S. arvensis*), SAR6 (*S. arvensis*), and DMU2 (*D. muralis*) had 7.12, 7.75, and 10.25% of isofucosterol, respectively, (Table 3), which was 7-fold higher than in the commercial cultivars (0.94 ± 0.33%). High campesterol was observed in all four *H. incana* genotypes HIN29, HIN23, HIN20, and HIN37 at 14.56, 16.29, 17.06, and 22.38%, respectively, where the average amount of campesterol in the commercial cultivars was 9.82 ± 1.25%. The accessions SARS2 (*S. arvensis*) and UPM6563 (*B. incana*) also exhibited high campesterol at 14.90 and 19.47%, respectively. These accessions with high campesterol had lower sitosterol and isofucosterol. There was a single accession of *B. fruticulosa* (BFR6) that showed extremely low campesterol (2.5%), with corresponding high sitosterol. DMU2 (*D. muralis*), also another single accession, had a high percentage of both cholesterol (5.38%) and 24-methylcholesterol (1.12%).

In this study, 20 wild accessions belonging to 10 different species were used. Three species, *H. incana, S. alba* and *S. arvensis,* had multiple (at least four) genotypes analysed. There was still a larger variation observed between these wild accessions of the same species compared to the *B. napus* cultivars (Table 2). However, in comparison, accessions of the same species exhibited some similar trends. Eighteen *B. napus* cultivars had an average of 9.82% campesterol, with a small (1.25%) deviation among all cultivars. All five *S. arvensis* accessions had an average of 11.59% campesterol with a deviation of 2.24% whereas all four accessions of *H. incana* compared to other species in this study had high campesterol at 14.56, 16.29, 17.06, and 22.38% (Table 3), and all *S. alba* genotypes exhibited low campesterol levels at 5.42, 6.43, 6.06, and 7.11% (Table 3). Another similar trend was comparatively higher isofucosterol in all *S. arvensis* accessions at 3.24, 3.68, 4.03, 7.17, and 10.27% (Table 3). For all other phytosterols, there were no similar trends observed within the same species.

### 2.2. Phytosterol Ratio Variation in Genotypes

**Figure 1:**
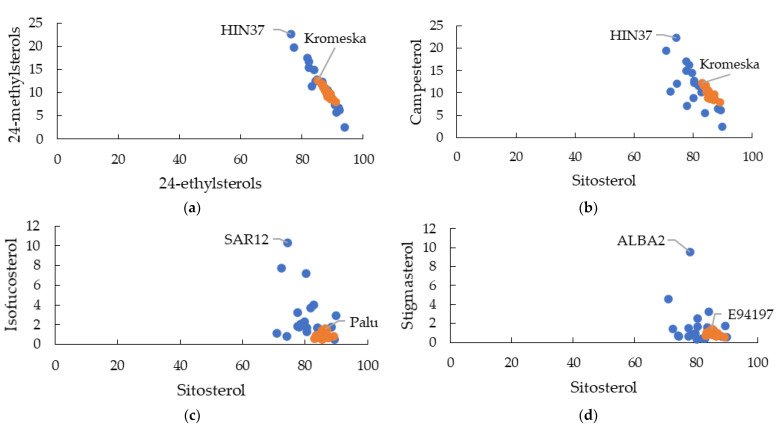
Comparison of the phytosterol ratios of (**a**) 24-methylsterols to 24-ethylsterols, (**b**) campesterol to sitosterol, (**c**) isofucosterol to sitosterol, and (**d**) stigmasterol to sitosterol among the commercial (orange) and wild (blue) accessions. Wild accessions showed higher ratio diversity. Those labelled are wild accessions with the highest phytosterol ratio that can influence insect feeding behaviour and stresses responses.

The ratios between each individual phytosterol were analysed by mapping scatter plots for all genotypes. As expected, wild accessions had a higher ratio diversity between all phytosterols compared to the *B. napus* commercial cultivars. Scatter plots for the ratio among the major phytosterols are shown in Figure 1. Some wild accessions exhibited higher phytosterol ratios than the other genotypes. The wild accession HIN37 (*H. incana*) showed twice as high a ratio for 24-methylsterols (campesterol and 24-methylcholesterol) to 24-ethylsterols (sitosterol, isofucosterol, stigmasterol, sitostanol, and stigmast-7-enol) (Figure 1a) and campesterol to sitosterol (Figure 1b) compared to the *B. napus* commercial cultivars. The wild accession SAR12 (*S. arvensis*) had a 7-fold higher isofucosterol to sitosterol ratio (Figure 1c) and ALBA2 (*S. alba*) had a 6-fold higher stigmasterol to sitosterol ratio (Figure 1d) compared to the average in the *B. napus* commercial cultivars.

### 2.3. Phytosterol Correlation

There was a highly significant (*p* < 0.001) inverse correlation (r = −0.97) observed between the 24-methylsterol and 24-ethylsterol compositions among all the plant samples (Table 4). Campesterol and sitosterol showed a highly significant (*p* < 0.001) correlation to 24-methylsterols and 24-ethylsterols (r = 1.00 and r = 0.82), being major constitutes of the latter, respectively. Correlations between the individual phytosterols can be seen in Table 4.

### 2.4. B. napus Leaf and Seed Phytosterol Comparison

**Figure 2:**
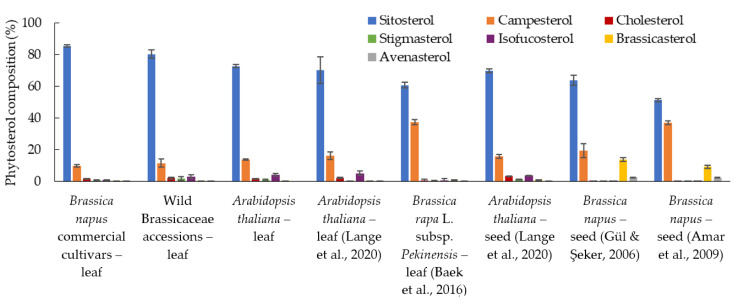
Comparison of the average phytosterol profiles from the seed and leaf samples of *B. napus* commercial cultivars, related wild accessions, and some other Brassicaceae species [24,25,27,28].

This was the first phytosterol study from the leaf samples of *B. napus* genotypes and other closely related wild accessions. Phytosterol profile data from two previous studies [24,25] that analysed seed phytosterol profiles from 19 and 27 *B. napus* cultivars (grown in field conditions) was used for a comparison with our leaf phytosterol profiles (Figure 2). There were major differences observed between the *B. napus* seed and leaf phytosterol profiles. The accumulation of 24-methylsterols was significantly higher in the *B. napus* seed compared to the leaf samples, consequently, there were lower 24-ethylsterols in the seed samples compared to the leaf samples (Figure 2). In the *B. napus* seed samples grown in the same field conditions [24], the average 24-methylsterols (campesterol and brassicasterol) was reported to be 19.32 and 13.90% respectively, compared to the *B. napus* leaf samples, which contained 9.84% and <0.02%, respectively. The accumulation of the 24-ethylsterols (sitosterol, stigmasterol, avenasterol, and isofucosterol) in seeds was 63.92%, 0.26%, 2.44% and not-reported compared to 85%, 0.95%, <0.02%, and 0.94% in the leaf samples, respectively. Avenasterol and brassicasterol were two predominant phytosterols found in the *B. napus* seed samples that were not significantly (>0.02% of total phytosterol) present in the leaf samples.

### 2.5. B. napus Leaf Phytosterol Profile Comparison with Related Plants

The phytosterol profile data from other Brassicaceae plant leaf sample studies [27,28] were included to establish the phytosterol profile differences between the related plant species (Figure 2). Phytosterol profiles from *B. napus* in comparison to *A. thaliana* leaf showed lower campesterol and isofucosterol; 9.8 and 0.94% in *B. napus* compared to 13.7 and 4.3% in *A. thaliana* (Figure 2). The reason for including *A. thaliana* in our study was for a comparison with the *B. napus* leaves and to compare our phytosterol quantification method with other studies. Our study reported a similar phytosterol profile for *A. thaliana* leaf samples compared to a recent study [27] that employed the same growth conditions and quantification method (Figure 2). In the leaf to leaf profile comparison with one of the closest possible related species, *B. rapa* L. subsp. *Pekinensis*, some dissimilarities were observed (Figure 2). Mainly, the *B. rapa* L. subsp. *Pekinensis* 11-week old leaf samples [28] had higher campesterol and lower sitosterol compared to the 2-week old *B. napus* leaf samples (Figure 2). Moreover, brassicasterol, a characteristic sterol of the Brassicaceae family, was absent in the *B. napus* leaf samples, however, a small amount of brassicasterol (0.73%) was found in the *B. rapa* L. subsp. *Pekinensis* leaves [28].

### 2.6. Low Phytosterol Variation in B. napus Genotypes Compared to Related Studies

**Figure 3:**
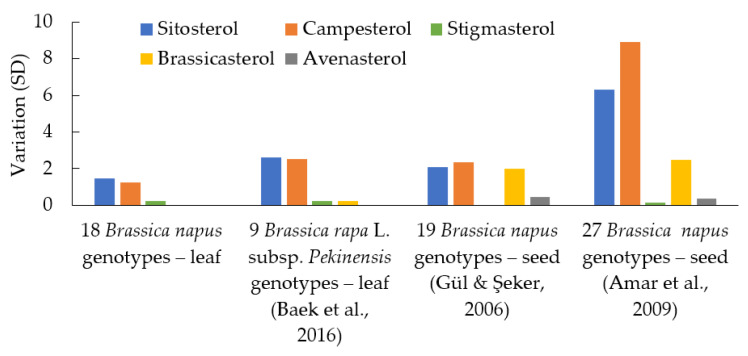
Comparison of the *B. napus* (leaf samples) phytosterol variation among genotypes with the *B. rapa* L. subsp. *Pekinensis* (leaf samples) genotypes and *B. napus* commercial cultivars (seed samples) grown in field conditions [25,28,29].

Phytosterol data from similar studies [24,25,28] were used to compare the variation among genotypes (Figure 3). There was some degree of sterol variation observed between the 18 genotypes of the *B. napus* cultivars (Table 1, Figure 3). However, the variation in the *B. napus* genotypes in this study was lower than that found in the 11-week old leaf samples from nine *B. rapa* genotypes [28] using similar growth conditions and seed samples from 19 genotypes [24] and 27 genotypes [25] grown in field conditions (Figure 3).

## 3. Discussion

This is the first-time that phytosterol variation among *B. napus* genotypes from leaf samples has been reported. Previously, phytosterol profiles from the seeds had only been characterised. Unlike in *A. thaliana* (a Brassicaceae plant family member), where two organs have a similar phytosterol profile, major differences were found between our leaf results with the previously reported seed sample profiles [24,25]. The higher composition of 24-ethylsterols (sitosterol, isofucosterol, stigmasterol, avenasterol, sitostanol, and stigmast-7-enol) and lower composition of 24-methylsterols (campesterol, brassicasterol, and 24-methylcholesterol) observed in our leaf results compared to the *B. napus* seeds might be due to the higher expression of *SMT2* (sterol methyltransferase 2) in the leaves. SMT2 is known to add methylation at the C24 carbon controlling the 24-methysterol to 24-ethysterol ratio in *A. thaliana* [48]. Moreover, avenasterol was also absent in the *B. napus* leaves, however, isofucosterol was present instead. Avenasterol is a precursor of 5-dehydroavenasterol, which is a precursor to isofucosterol. This suggests a higher expression of *DWF7* (C-5 sterol desaturase) and *DWF5* (sterol delta7 reductase) genes in the leaves compared to the seeds. Subsequent action of DWF7 and DWF5 is responsible for the avenasterol into isofucosterol conversion [49]. Finally, the characteristic Brassicaceae family sterol, brassicasterol, was abundantly (13%) found in *B. napus* seeds [24] and present in a small quantity (0.73%) in the *B. rapa* leaves [28], whereas it was absent in our *B. napus* leaves. Future studies could investigate the sterol synthesis gene expression in *B. napus* tissues.

In the related species leaf to leaf profile comparison (Figure 2), no major differences were observed between the *B. napus* and *A. thaliana* leaf phytosterol profiles. The most notable difference between the *B. napus* and *B. rapa* L. subsp. *Pekinensis* profiles was a comparatively higher campesterol composition in *B. rapa* L. (Figure 2). The campesterol to sitosterol ratio has been associated with cell elongation regulation during growth in cotton [50], hence, part of the high campesterol composition differences could have been contributed by the growth activity in the tissue samples.

The phytosterol variation among the *B. napus* genotypes in the leaf samples in this study was lower compared to that reported in the *B. napus* seed and *B. rapa* L. subsp. *Pekinensis* leaf studies [24,25,28] (Figure 3). Phytosterol variation (SD) among the major phytosterols was 3-fold lower in our *B. napus* genotypes grown in a controlled environment compared to the 27 *B. napus* genotypes (seed samples) grown in six variable field condition locations [25]. This higher phytosterol variation was due to high environmental effect because of six variable field conditions. Phytosterol profiles are heavily affected by planting location and environmental conditions [26]. There was also less than 1-fold lower phytosterol variation (SD) compared to the 19 *B. napus* cultivars (seed samples) [24] grown in one field condition location. In this study, the plants were only grown for two weeks for the true leaves to fully develop, with no additives and in a controlled environment (phytotron), which had far less of an environmental effect. This could be a reason for our *B. napus* genotypes having less phytosterol variation compared to the field studies. Compared to nine *B. rapa* L. subsp. *Pekinensis* (leaf sample) genotypes [28] also grown in a controlled environment, the phytosterol variation among our *B. napus* genotypes was still lower (Figure 3). In our 18 *B. napus* and the nine *B. rapa* L. subsp. *Pekinensis* [28] genotype study, both had a small sample size, so larger scale studies are needed to establish lower phytosterol variation in the *B. napus* cultivars due to genotypic effect, which could be due to the loss of genetic diversity during decades of domestication in modern *B. napus* cultivars [34].

This study focused on investigating the potential of genotypes producing a novel composition of phytosterols that can be used to generate plants with the ideal phytosterol profiles. Hence, phytosterol composition was the emphasis, and the results are reported as composition percentages rather than the absolute content of the individual phytosterol. The total phytosterol content (TPC) is considered as a separate trait compared to genes and genetic factors controlling the conversions of phytosterols during the melovate pathway [4]. In a *B. napus* genome wide association study [30], 11 quantitative trait loci (QTL) were found to be responsible for the variation in composition among the major phytosterols in oilseeds of *B. napus*, and only one minor QTL overlapped with TPC. Moreover, in previous studies, no direct correlation has been found between a composition percentage of individual phytosterols and TPC [24,25].

There has been growing interest in studying the phytosterol composition from plant leaves. Novel phytosterol profiles, which include novel phytosterols and phytosterol ratios, have implications in plant protection from insect attack. This study further explores the genotypic variation of phytosterol profiles in the leaf samples of *B. napus* cultivars and related wild Brassicaceae accessions as a potential for acquiring insect tolerance, perhaps through breeding. Phytosterol diversity in wild accessions could be the key in obtaining novel sterols and sterol ratios for insect resistance. Since cholesterol is essential for the growth and development of herbivore insects relying on converting host phytosterols to cholesterol [51], there are stringent structural demands on phytosterols used as substrates [20].

Among the eight phytosterols detected in this study from leaf samples, sitosterol is the most suitable phytosterol for the most common herbivorous insect species to convert into cholesterol. A total of 47 out of 60 insect species fed on individual sitosterol had good growth and body mass [20], whereas stigmasterol, an unsuitable phytosterol, when fed upon, 43 out of 59 common insect species had no or weak growth [20]. Another two major sterols found in our *B. napus* leaf samples, campesterol and isofucosterol (Table 1), have not been studied in a large number of species. However, campesterol feeding studies showed weak growth of house fly (*Musca domestica*) and honeybee (*Apis mellifera*), but promoted growth in a moth (*Manduca sexta*) [52,53,54]. Moreover, in transgenic lines of *A. thaliana*, increasing the campesterol to sitosterol ratio from 2:8 to 4:5 using sterol-C24-methyltransferase 2 co-suppressor lowered the aphids’ (*M. persicae*) body mass and pupation number. In the same study, increasing the isofucosterol to sitosterol ratio in the transgenic *A. thaliana* ratio from 2:8 to 4:6 using mutant cycloartenol synthase 1 significantly reduced the growth and pupation rate of aphids (*M. persicae*) [12]. Since campesterol and isofucosterol are non-predominant phytosterols, more insect feeding studies are needed to establish their non-suitability in different insect pest species. Moreover, the genotypes with high levels of campesterol, isofucosterol, or stigmasterol had consequently lower amounts of the most predominant and common suitable sterol, sitosterol.

Mixed diet insect feeding studies have established that phytosterol profiles with enough suitable sterol to support insect growth and development can still gain insect resistance by having high levels of unsuitable sterol(s) present in the same diet [21]. Hence, the utilisation of suitable sterols by insects can be hindered by increasing the ratio of unsuitable sterols. If the ratio of unsuitable to suitable phytosterols reaches a certain threshold, it affects the insect’s growth and development [21]. Currently, the small number of mixed sterol diet feeding studies makes it difficult to suggest ideal sterol profiles for broad species insect tolerance. In our study, the highest unsuitable to suitable phytosterol ratio (stigmasterol+ Isofucosterol+ campesterol: sitosterol+ cholesterol) was 1:9 in the cultivar Kromeska, which was similar to the average ratio of *B. napus* commercial cultivars (Supplementary Figure S2), whereas in two wild accessions, the unsuitable to suitable phytosterol ratio was 3:7 in HIN37, with the second highest of 2:7 in UPM6563 (Supplementary Figure S2). This might not be enough to gain insect resistance [12,55]. Individually, three different wild accessions had a high composition of unsuitable individual phytosterols, HIN37 (*H. incana*), ALBA2 (*B. tournefortti*), and SAR12 (*S. arvensis*), with the highest isofucosterol, stigmasterol, and campesterol, respectively (Table 3). For three wild accessions, the ratio of campesterol, isofucosterol, and stigmasterol to sitosterol was 2-fold, 6-fold, and 7-fold higher compared to the commercial accessions, respectively (Supplementary Figure S2). The growth and development struggles of insects rendered on wild Brassicaceae species [35,36,37,38,39,40,41,42,43,44,45] can be from an accumulative effect contributed by several plant chemicals such as glucosinolates, saponins, flavonoids, and unsuitable phytosterols [32,35]. However, functional studies have established that insect resistance can be established solely based on the phytosterol profiles [12]. The key strategy to gaining environmentally friendly insect resistance is to convert the common predominant suitable phytosterols (sitosterol) into other unsuitable phytosterols according to the targeted insect. Hypothetically, accumulating high amounts of individual unsuitable sterols present in wild accessions through breeding in a single cultivar could have a good chance of gaining insect resistance. A larger scale of study in wild accessions to explore rarer and greater phytosterol variations would be useful.

In our study, another important ratio in the leaf samples was stigmasterol to sitosterol. In this study, the average ratio of stigmasterol to sitosterol in commercial accessions was 1:86 (Figure 1d). A single wild accession, ALBA 2, exhibited 10-fold higher stigmasterol with a 1:8 stigmasterol to sitosterol ratio (Figure 1d). However, it was far less than that observed in the temperature tolerant *A. thaliana* overexpressing CYP710A1 [8]. Higher levels of stigmasterol levels also contributed to enhanced resistance to bacterial pathogen infection [11] and enhanced drought tolerance [23] whereas the effect of a smaller increase in the stigmasterol ratio has not been studied.

Wild accessions from this study showed the promise of having a large genetic diversity for phytosterol profiles compared to the *B. napus* commercial cultivars. This may be due to decades of domestication in modern *B. napus* cultivars [34], leading to significant gene pool loss. Diversity loss in commercial cultivars has been previously described [56]. Presently, marker assisted breeding is considered as the most prominent technique of acquiring traits [57]. This study shows the potential of wild accessions to be used to acquire to ideal phytosterol ratios, which could help plants in response to stresses and insect attack, however, further studies investigating the ratios in more accessions and species are required.

## 4. Materials and Methods

### 4.1. Plant Material

Eighteen commercial cultivars of *B. napus* and 20 wild Brassicaceae accessions from 10 species (*B. fruticulosa*, *B. incana*, *B. macrocapa*, *B. montana*, *Camelina sativa*, *D. muralis*, *H. incana*, *Raphanus sativus*, *S. alba*, *S. arvensis*) were used in this study (Table 5). The commercial cultivars selected had variable geographic origins to provide a wide representation of the commercially cultivated gene pool. *A. thaliana* was also included for comparison and standardization purposes.

### 4.2. Growth Conditions

*Brassica napus* and wild Brassicaceae accessions were grown simultaneously in a controlled phytotron environment. Eighteen commercial cultivars of *B. napus* and 20 wild Brassicaceae accessions from 10 species (*B. fruticulosa*, *B. incana*, *Brassica macrocapa*, *Brassica montana*, *Camelina sativa*, *D. muralis*, *H. incana*, *Raphanus sativus*, *S. alba*, *S. arvensis*) were used in this study (Table 5). The commercial cultivars selected had variable geographic origins to provide a wide representation of the commercially cultivated gene pool. All the above-mentioned plant material was grown simultaneously in a controlled phytotron environment. Six seeds were sown for each genotype in 7 cm deep plastic trays with 48-cell inserts (cell size dimensions 5.7 cm × 3.8 cm and 5.4 cm deep), one seed per cell. Normal, ground dug, sterile potting mix was used with no additives. Growth conditions were set as follows: temperature at 22 °C, humidity at ~50%, and photoperiod of 12 h light (under sunlight) and 12 h dark (covered). In addition, *A. thaliana* was grown separately in a fully controlled environment using a Conviron Adaptis^®^ growth cabinet. The potting mix used was composed of a 3:1:1 ratio of soil, perlite, and vermiculite, respectively. *A. thaliana* seeds were germinated in soil (6 × 6 cm^2^ pots) and placed in a growth cabinet with the environmental conditions set as follows: temperature 22 °C, relative humidity (RH) 70%, and 16 h day/8 h night photoperiod with 120 prf light intensity at the soil surface level. Two leaf tissue samples of 300 mg of tissue material were collected from all plants. Samples were stored in 2 mL plastic tubes at −30 °C until use.

### 4.3. Sterol Extraction

The phytosterol extraction process was adapted from [58]. Six replicates were analysed for each genotype. For alkaline hydrolysis, 1 M methanolic KOH was prepared with 2% potassium hydroxide in methanol and water (3:2). A total of 1 mL of this solution was vortexed with 300 mg of leaf material for alkaline hydrolysis and kept at 70 °C for 2 h. After reaching room temperature, for the extraction of non-saponifiable matter, 500 µL of hexane was added and vortexed for 20 s and centrifuged at 8000× *g* for 1 min. The supernatant (clearly separated upper yellowish layer) was carefully pipetted out of the tube and transferred into a fresh 1.5 mL tube. This step was repeated to make sure that all of the free phytosterols were extracted. Later, for the evaporation of hexane, samples were placed in a fume safety cupboard (LabAire™) overnight at 37.5 °C with a continuous nitrogen (N_2_) stream flow. The dried samples were stored at room temperature until the GC-MS was performed.

### 4.4. Gas Chromatography and Mass Spectrometry

Samples were prepared for GC-MS (gas chromatography and mass spectrometry) analysis by adding 5 μL of pyrinde and 5 μL of BSTA to dry phytosterol samples. The samples were vortexed for 15 s and 40 μL of chloroform was added. The solution was transferred to a 50 μL GC vial and 1 μL was injected into the GC-MS (Agilent GC 6890N gas chromatograph fitted with a 7683B Automatic Liquid Sampler and a 5975B Inert MSD quadrupole MS detector (Agilent Technologies). The capillary column on the gas chromatograph was 0.25 mm (i.d.) with a 0.25 µm film thickness, and 30 m Varian FactorFour VF-5ms and was fitted with a 10 m integrated guard column (Varian). The inlet temperature was constant at 300 °C. The helium carrier gas flowed at a constant rate of 1 mL min^−1^. The GC oven temperature was set at 100 °C initially for 1 min with an increase to 320 °C at a rate of 37 °C min^−1^ and then held for 2 min. The transfer accession temperature was set at 280 °C, MS source at 230 °C, and quadrupole temperature at 150 °C. Ionization was by electron impact at 70 eV. The mass calibrant perfluorotributylamine was used to pre-tune the MS. Data analysis was performed through Agilent GC/MSD Productivity Chemstation software. The phytosterols were identified by comparing their retention times and mass to that of the internal standard.

### 4.5. Statistical Analysis

Calculations of the averages, standard deviation, ratios, and correlations relied on SPSS Statistics for Windows, Version 28.0 Released 2021 IBM Corporation. Charts, scatter plots, and the histogram were plotted in spreadsheets using Microsoft Excel Version 2202 Build 16.0.14931.20118.

## 5. Conclusions

In conclusion, from this study, we found that the phytosterol accumulation and diversity between the seeds and leaf samples were inconsistent. The ratio of 24-ethysterols to 24-methylsterols was found to be higher in the *B. napus* leaves, and the overall phytosterol diversity in the *B. napus* commercial cultivars was found to be significantly lower compared to previous field condition oilseed studies [24,25]. We also discovered that there was a high phytosterol variation in the wild Brassicaceae accessions compared to the commercial cultivars. Some highlighted wild accessions in this study exhibited novel sterol compositions and ratios of stigmasterol, campesterol, and isofucosterol as well as the ratio to sitosterol as they are of importance. These wild accessions show promise for breeding purposes to achieve some extent of the crop improvement traits proposed by the phytosterol functional studies whereas a larger set of wild species could also result in more interesting phytosterol profiles. Moreover, further functional studies are needed to study the effect of variable sterol ratios in crops to establish the further significance of these wild accessions. The strategy employed in this study has the potential to be applied to a broad range of agricultural crops such as rice, barley, and wheat for crop protection.

## Figures and Tables

**Table 1:** Comparison of the composition of eight major phytosterols in *B. napus* commercial cultivars (Cm) and Brassicaceae wild accessions (Wl). Minor phytosterols present in trace amounts of less than 0.02% are not reported. The mean (average), range, and standard deviation (SD) of the phytosterol percentages are presented.

Phytosterols	Average (%)		Range (%)		SD (%)	
	Cm	Wl	Cm	Wl	Cm	Wl
24-Methylcholesterol	0.26	0.36	0.11–0.52	0.13–1.12	0.12	0.21
Cholesterol	1.79	1.89	1.09–2.43	0.93–5.38	0.42	1.11
Campesterol	9.82	11.5	7.86–12.26	2.50–22.38	1.25	4.94
Isofucosterol	0.94	2.93	0.51–1.61	0.50–10.27	0.33	2.58
Sitostanol	0.44	0.63	0.20–0.69	0.31–1.87	0.13	0.37
Sitosterol	85.55	80.44	83.07–89.1	70.91–89.90	1.49	5.29
Stigmast-7-enol	0.24	0.34	0.10–0.41	0.15–0.52	0.10	0.10
Stigmasterol	0.95	1.73	0.58–1.45	0.30–9.57	0.25	2.13

**Table 2:** Variance of the major phytosterols (>0.02% of total phytosterol); 24-methylcholesterol (mchol), cholesterol (chol), campesterol (cam), stigmasterol (stigm), isofucosterol (ifuc), sitostanol (sitn), stigmas-7-tenol (stig7), and sitosterol (sitos) distribution between 18 genotypes of *B. napus* commercial cultivars.

Names	MCHOL	CHOL	CAM	IFUC	SITN	SITOS	STIG7	STIGM
Alaska	0.52	2.07	8.76	1.19	0.37	85.62	0.15	1.32
Beluga	0.11	1.15	7.86	0.84	0.20	89.14	0.11	0.58
Darmor	0.17	1.40	9.42	1.04	0.53	86.05	0.30	1.09
Dippes	0.31	1.92	10.09	0.95	0.68	84.54	0.31	1.20
E94197	0.46	2.34	8.93	0.90	0.69	85.04	0.19	1.45
Fortin	0.14	1.96	8.50	0.85	0.35	87.34	0.18	0.68
GLuesewitzer	0.39	1.79	11.54	0.92	0.56	83.46	0.32	1.01
Kromerska	0.42	2.25	12.26	0.56	0.51	83.07	0.21	0.72
Major	0.16	1.89	11.06	0.65	0.58	84.33	0.17	1.17
Olimpiade	0.18	1.50	11.74	0.76	0.43	84.31	0.20	0.89
Pacific	0.17	2.03	10.57	0.58	0.35	85.11	0.10	1.08
Palu	0.26	1.44	8.80	1.61	0.35	86.51	0.37	0.67
Pirola	0.22	2.13	9.82	0.51	0.54	85.55	0.41	0.82
R53	0.17	1.50	10.52	0.93	0.45	85.28	0.39	0.77
Rapid	0.26	2.05	9.97	1.06	0.31	84.91	0.27	1.16
Savannah	0.28	2.43	8.90	1.45	0.41	85.43	0.29	0.81
Vivol	0.11	1.09	9.60	0.67	0.38	87.31	0.12	0.73
Wotan	0.26	1.23	8.44	1.55	0.31	86.96	0.31	0.94
Mean	0.26	1.79	9.82	0.94	0.44	85.55	0.24	0.95
Minimum	0.11	1.09	7.86	0.51	0.20	83.07	0.10	0.58
Maximum	0.52	2.43	12.26	1.61	0.69	89.14	0.41	1.45
SD	0.12	0.42	1.25	0.33	0.13	1.49	0.10	0.25

**Table 3:** Variance of major phytosterols (>0.02% of total phytosterol); 24-methylcholesterol (mchol), cholesterol (chol), campesterol (cam), stigmasterol (stigm), isofucosterol (ifuc), sitostanol (sitn), stigmas-7-tenol (stig7), and sitosterol (sitos) distribution between 20 genotypes of wild Brassicaceae accessions.

Names	Specie	MCHOL	CHOL	CAM	IFUC	SITN	SITOS	STIG7	STIGM
ALBA 2	*Sinapis alba*	0.33	1.93	7.11	1.77	0.77	78.07	0.45	9.57
ALBA 17	*Sinapis alba*	0.31	1.02	5.42	1.65	1.87	84.04	0.39	3.20
BFR 6	*Brassica fruticulosa*	0.13	3.42	2.50	2.95	0.31	89.90	0.26	0.54
CLARO	*Raphanus sativus* L.	0.23	3.00	12.28	1.72	0.40	80.44	0.25	1.68
DMU 2	*Diplotaxia* *muralis*	1.12	5.38	10.30	7.75	1.20	72.34	0.47	1.43
FRITSCH CAME	*Camelina sativa*	0.14	1.63	10.84	0.94	0.80	83.76	0.33	1.57
HIN 20	*Hirschfeldia incana*	0.38	0.93	17.06	1.87	0.38	77.62	0.27	1.48
HIN 23	*Hirschfeldia incana*	0.47	0.99	16.29	2.04	0.37	78.67	0.37	0.80
HIN 29	*Hirschfeldia incana*	0.41	1.09	14.56	2.33	0.50	79.75	0.42	0.94
HIN 37	*Hirschfeldia incana*	0.23	0.97	22.38	0.82	0.42	74.23	0.24	0.71
PI 284858	*Sinapis alba*	0.19	1.54	6.06	0.50	0.41	89.41	0.15	1.75
PI 312848	*Sinapis alba*	0.30	1.34	6.43	1.76	0.91	88.32	0.34	0.60
SAR 12	*Sinapis arvensis*	0.41	0.99	12.01	10.27	0.67	74.48	0.52	0.64
SAR 2	*Sinapis arvensis*	0.47	2.43	14.90	3.24	0.44	77.59	0.30	0.64
SAR 3	*Sinapis arvensis*	0.44	1.25	11.59	3.68	0.45	81.85	0.35	0.38
SAR 5	*Sinapis arvensis*	0.41	1.26	10.14	4.03	0.63	82.83	0.31	0.40
SAR 6	*Sinapis arvensis*	0.51	1.93	8.81	7.17	0.61	80.17	0.50	0.30
UPM 3819	*Brassica macrocapa*	0.27	1.65	10.73	1.70	0.40	83.95	0.38	0.93
UPM 6563	*Brassica incana*	0.25	2.91	19.47	1.10	0.56	70.91	0.25	4.55
UPM 6813	*Brassica montana*	0.21	2.19	12.66	1.30	0.41	80.41	0.25	2.56
Mean		0.36	1.89	11.58	2.93	0.63	80.44	0.34	1.73
Minimum		0.13	0.93	2.50	0.50	0.31	70.91	0.15	0.30
Maximum		1.12	5.38	22.38	10.27	1.87	89.90	0.52	9.57
SD		0.21	1.11	4.94	2.58	0.37	5.29	0.10	2.13

**Table 4:** Coefficient correlation (r) for phytosterols; 24-methylcholesterol (mchol), cholesterol (chol), campesterol (cam), stigmasterol (stigm), isofucosterol (ifuc), sitostanol (sitn), stigmast-7-tenol (stig7), sitosterol (sitos), and 24-methylsterols (mestl) in 18 *B. napus* commercial cultivars and 20 related wild Brassicaceae accessions. *, **, *** represents significance at the 0.05, 0.01, 0.001 level, respectively.

Phytosterols	MCHOL	CHOL	CAM	IFUC	SITN	SITOS	STIG7	STIGM	MESTL
Cholesterol	0.32 ***								
Campesterol	−0.07	0.09							
Isofucosterol	0.18 *	0.27 ***	−0.05						
Sitostanol	0.35 ***	0.23 **	0.02	0.24 ***					
Sitosterol	−0.32 ***	−0.32 ***	−0.74 ***	−0.45 ***	−0.33 ***				
Stigmast-7-enol	0.08	0.38 ***	−0.05	0.37 ***	0.08	−0.19 **			
Stigmasterol	0.20 **	−0.02	0.04	−0.09	0.20 **	−0.38 ***	−0.03		
24-methylsterols	−0.05 ***	0.15	1.00 ***	−0.03	0.03	−0.76 ***	−0.02	0.03	
24-ethylsterols	−0.19 **	−0.23 **	−0.96 ***	−0.01	−0.12	0.82 ***	0.00	−0.08	−0.97 ***

**Table 5:** The Brassicaceae genotypes utilized in this study.

S. No.	Name	Species	S. No	Name	Species
1	Alaska	*Brassica napus*	21	Olimpiade	*Brassica napus*
2	ALBA17	*Sinapis alba*	22	Pacific	*Brassica napus*
3	ALBA2	*Sinapis alba*	23	Palu	*Brassica napus*
4	Beluga	*Brassica napus*	24	PI284858	*Sinapis alba*
5	BFR6	*Brassica fruticulosa*	25	PI312848	*Sinapis alba*
6	CLARO	*Raphanus sativus* L.	26	Pirola	*Brassica napus*
7	Col-0	*Arabidopsis thaliana* (Ecotype Columbia)	27	R53	*Brassica napus*
8	Darmor	*Brassica napus*	28	Rapid	*Brassica napus*
9	Dippes	*Brassica napus*	29	SAR12	*Sinapis arvensis*
10	DMU2	*Diplotaxia muralis*	30	SAR2	*Sinapis arvensis*
11	E94197	*Brassica napus*	31	SAR3	*Sinapis arvensis*
12	Fortin	*Brassica napus*	32	SAR5	*Sinapis arvensis*
13	FRITSCH CAME	*Camelina sativa*	33	SAR6	*Sinapis arvensis*
14	GLuesewitzer	*Brassica napus*	34	Savannah	*Brassica napus*
15	HIN20	*Hirschfeldia incana*	35	UPM3819	*Brassica macrocapa*
16	HIN23	*Hirschfeldia incana*	36	UPM6563	*Brassica incana*
17	HIN29	*Hirschfeldia incana*	37	UPM6813	*Brassica. montana*
18	HIN37	*Hirschfeldia incana*	38	Vivol	*Brassica napus*
19	Kromerska	*Brassica napus*	39	Wotan	*Brassica napus*
20	Major	*Brassica napus*			

## Data Availability

Data can be found in the M.B. Master program thesis at the University of Western Australia, Australia.

## Supplementary Materials

The following supporting information can be downloaded at: https://www.mdpi.com/article/10.3390/plants12091866/s1, Figures S1: Comparison of selected genotypes with high phytosterol ratios in *Brassica napus* commercial cultivars and a Brassicaceae wild accession.; Figure S2: Comparison of selected genotypes with high suitable to unsuitable phytosterols ratio for insect resistance.
