# Supplementary material for: Diversity of Phytosterols in Leaves of Wild Brassicaceae Species as Compared to Brassica napus Cultivars: Potential Traits for Insect Resistance and Abiotic Stress Tolerance
Source: Plants (Basel). 2023 May 1;12(9):1866. doi: 10.3390/plants12091866 (PMC10180710; doi:10.3390/plants12091866)
Supplement: Supplementary file 1 [file plants-12-01866-s001.zip › plants-2272693-supplementary.pdf]

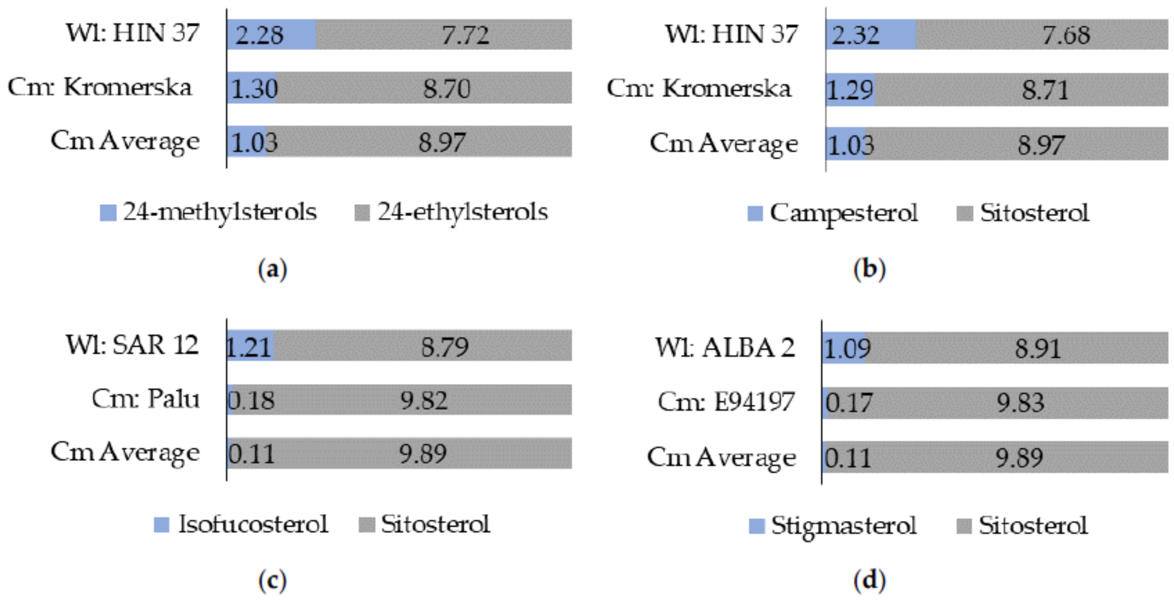

Figure S1: Comparison of selected genotypes with high phytosterol ratios in Brassica napus commercial cultivars and a Brassicaceae wild accession. (**a**) 24-methylsterols to 24-ethylsterols, (**b**) campesterol to sitosterol, (**c**) isofucosterol to sitosterol and (**d**) stigmasterol to sitosterol. Average ratios found in commercial cultivars (Cm Average) are compared with genotypes with a high ratio in commercial (Cm) and a wild (Wl) accession. For each chart, the ratios between individual sterols are standardized out of 10 for comparison.

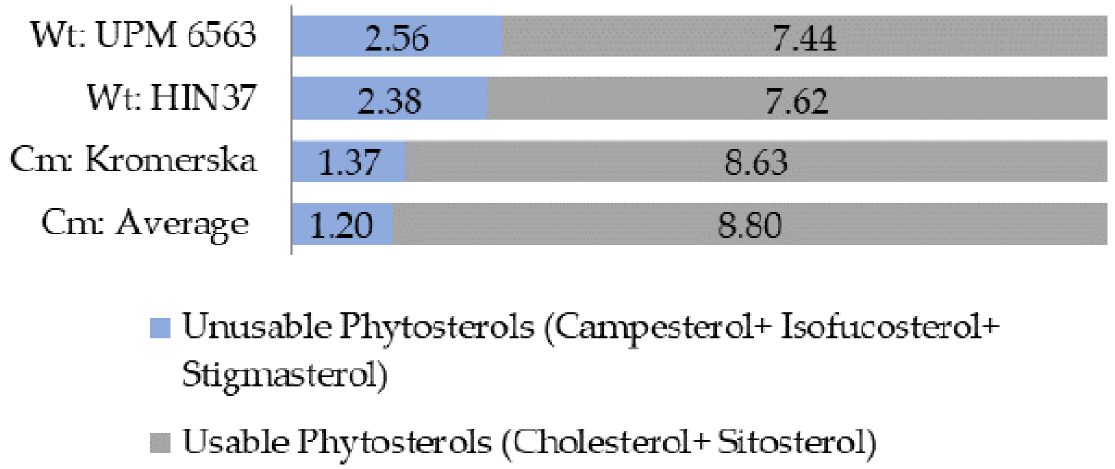

Figure S2: Comparison of selected genotypes with high suitable to unsuitable phytosterols ratio for insect resistance. Comparison of genotypes with high suitable (cholesterol and sitosterol) to unsuitable phytosterols (campesterol, isofucosterol and stigmasterol) ratio. Two of wild accessions (Wt: UPM3563 and HIN37) showed higher ratio as compared to highest commercial cultivar (Cm: Kromeska) and commercial average (Cm: Average). Suitable and unsuitable phytosterols are with respect to generally promoting growth and development in herbivore insect. Each ratio is standardized out of 10 for comparison.
